# Roles of Hospital-Based Gerontological Nurse Specialists in Supporting Integrated Care for Older Adults: A Scoping Review Protocol

**DOI:** 10.3390/ijerph23080968

**Published:** 2026-07-27

**Authors:** Jingwen Yang, Li Yao, Zhouyuan Peng, Kumiko Kuroda

**Affiliations:** 1 Department of Advanced Gerontological Nursing, Graduate School of Nursing, Chiba University, Chiba 260-0856, Japan; yaoli134193@chiba-u.jp (L.Y.); kkuroda@faculty.chiba-u.jp (K.K.); 2 Medical School, Lihu Campus, Shenzhen University, Shenzhen 518060, China; pengzhy@szu.edu.cn

**Keywords:** gerontological nurse specialist, nurse’s role, integrated care, older adults, advanced nursing practice, scoping review

## Abstract

**Highlights:**

**Public health relevance—How does this work relate to a public health issue?**

- Population ageing and increasingly complex care needs require integrated, person-centred health and social care for older adults.
- Hospital-based gerontological nurse specialists are well positioned to support integrated care through care coordination, transitional care, and continuity of care.

**Public health significance—Why is this work of significance to public health?**

- This protocol addresses the lack of synthesized evidence regarding the roles of hospital-based gerontological nurse specialists in supporting integrated care for older adults.
- The review will provide a comprehensive mapping of role conceptualizations, functions, responsibilities, and activities across different healthcare systems.

**Public health implications—What are the key implications or messages for practitioners, policy makers and/or researchers in public health?**

- The findings may inform role clarification, workforce development, nursing education, and future research on integrated gerontological care.
- The review may support the development of integrated care policies and healthcare services that promote continuity of care and ageing in place.

**Abstract:**

Background/Objectives: As population ageing accelerates, integrated care has become an important strategy for addressing the complex care needs of older adults. Although gerontological nurse specialists (GNSs) are expected to contribute to integrated care, their roles remain inconsistently defined and insufficiently synthesized across healthcare systems and professional role titles. This scoping review aims to systematically map the roles of GNSs in supporting integrated care for older adults within hospital-based settings. Methods: This scoping review protocol will follow the JBI methodology for scoping reviews and will be reported in accordance with the PRISMA-ScR guidelines. Searches will be conducted in PubMed, CINAHL, Web of Science, China National Knowledge Infrastructure, and Ichushi-Web, with no date restrictions. Studies published in English, Chinese, and Japanese will be included. The review will consider studies involving nurses with recognized specialist or advanced nursing practice competence in gerontological or older-adult care and will focus on their roles in supporting integrated care for older adults within hospital-based settings and hospital-linked transitional care services. Two independent reviewers will conduct study selection and data extraction. The findings will be synthesized descriptively and presented using tabular, narrative, and diagrammatic formats. Conclusions: This review is expected to contribute to a clearer understanding of the roles of GNSs in integrated care for older adults and provide evidence to inform future nursing practice, workforce development, education, and research in gerontological integrated care. Registration: This protocol was registered within the OSF database on 19 May 2026.

## 1. Introduction

### 1.1. The Global Imperative for Integrated Care for Older Adults

Older people currently constitute a larger proportion of the global population than ever before. In 2024, an estimated 1.18 billion people worldwide were aged 60 years or older, accounting for 14.5% of the global population, and this number is projected to reach 2.1 billion by 2050 [1]. Population ageing has been accompanied by a growing number of older adults living with complex health conditions, including multimorbidity, functional decline, frailty, and cognitive impairment [2]. These challenges frequently require care from multiple providers across different settings, making care coordination and continuity increasingly important [3].

In response to these challenges, integrated care has emerged as a leading approach for organizing and delivering services for individuals with complex, long-term needs [4]. Integrated care refers to health and social care services that are organized and delivered in a coordinated and person-centred manner, enabling individuals to receive a continuum of care across settings and throughout the life course [5]. Such care may include health promotion, disease prevention, diagnosis, treatment, rehabilitation, palliative care, and long-term care services [6,7]. Although interpretations of integrated care vary across countries and healthcare systems, a shared understanding is that integrated care seeks to reduce fragmentation in healthcare and social services through coordinated and continuous care delivery, thereby improving care experiences and health outcomes, particularly among populations with complex needs such as older adults [8,9,10].

Effective integrated care requires seamless connections across acute care, rehabilitation, community services, and long-term care settings. Previous research suggests that integrated care may reduce hospital admissions and lengths of stay among older adults [10], while also contributing to improved social participation and connectedness for older adults and their families [11]. In addition to improving person-centred outcomes, integrated care has become an important policy strategy for enhancing the sustainability of health and social care systems by reducing avoidable hospitalisations, preventing unnecessary institutionalization, and supporting older adults to age in place [4,12].

### 1.2. The Evolving Role of the Hospital in Integrated Care

Although integrated care is often discussed in the context of community-based services, hospitals remain important entry points for older adults with complex conditions and play a critical role in coordinating care across settings [4]. Hospitalization frequently initiates comprehensive geriatric assessment, multidisciplinary care planning, discharge planning, and referrals to rehabilitation, primary care, community services, and long-term care. Consequently, hospitals have evolved beyond their traditional role as providers of acute medical treatment and are increasingly recognized as collaborative partners, anchor institutions, and coordinators of care across health and social care sectors [13].

Within this continuum of care, hospitals play a particularly important role in facilitating care transitions. Effective transitional care begins during hospitalization and includes comprehensive discharge planning, patient and family preparation, medication reconciliation, communication among healthcare providers, and arrangements for post-discharge follow-up [14]. By coordinating these processes, hospitals function as a critical bridge between acute care and ongoing support in community and long-term care settings. Poorly managed transitions from hospital to home have been associated with adverse events, avoidable readmissions, and fragmented care, whereas high-quality transitional care contributes to continuity of care, safer recovery, and successful ageing in place [14,15]. These evolving responsibilities also highlight the importance of healthcare professionals who are capable of coordinating care across organizational boundaries and ensuring continuity throughout the care pathway.

### 1.3. Gerontological Nurse Specialists and Their Roles in Integrated Care

Nurses with specialist expertise in gerontological care are increasingly recognized as important contributors to integrated care for older adults [16,17,18,19,20]. As older adults often require services across multiple healthcare and social care settings, integrated care relies not only on system-level coordination but also on healthcare professionals capable of supporting continuity of care and communication across providers and services [21]. Within this context, gerontological nurse specialists (GNSs) may contribute through activities such as comprehensive assessment, care coordination, interdisciplinary communication, patient and family education, and facilitation of care transitions [18,19]. A qualitative study conducted in New Zealand found that both older adults and health professionals perceived GNSs as providing “holistic expertise” and enhancing communication, thereby helping reduce fragmentation and improve care coordination [22].

However, the terminology, scope, and structure of specialist nursing roles vary considerably across countries and healthcare systems. International nursing literature suggests that advanced practice nursing is not a universally standardized role category; rather, it encompasses different configurations of clinical nurse specialists, nurse practitioners, and other specialist or advanced nursing roles depending on local regulations, educational systems, and service models [23,24]. The International Council of Nurses (ICN) therefore provides a broad framework for advanced nursing practice while recognizing that role titles and scope of practice remain context dependent.

Although advanced practice nurses (APNs) assume diverse responsibilities across healthcare systems, ranging from independent clinical decision-making to facilitating communication and coordination between services and communities, they make important contributions to person-centred integrated care for older adults [20]. Accordingly, the present review adopts an operational definition of GNSs. GNSs are defined as hospital-based nurses with recognized specialist or advanced practice competence in older-adult care, including clinical nurse specialists (CNSs), APNs, nurse practitioners (NPs), or equivalent gerontological specialist roles, provided that their roles explicitly involve the care of older adults and activities related to integrated care processes.

This operational definition is necessary because the literature on specialist gerontological nursing remains heterogeneous and frequently uses different professional titles to describe overlapping functions and responsibilities. By defining GNSs according to functional role characteristics rather than professional titles alone, this review aims to capture the full range of specialist nursing contributions to integrated care for older adults while maintaining transparent and explicit inclusion boundaries.

### 1.4. Rationale for a Scoping Review

The roles of GNSs in supporting integrated care within hospital-based settings remain insufficiently synthesized in the literature. To the best of our knowledge, no previous scoping review or systematic review has specifically synthesized the roles of hospital-based GNSs in supporting integrated care for older adults across different healthcare systems. Although previous studies have examined advanced nursing roles in integrated care, the evidence remains fragmented and is frequently described using inconsistent terminology, varying role definitions, and diverse healthcare contexts [17,20]. Such heterogeneity limits a clear and systematic understanding of how GNS roles are conceptualized and enacted in practice, particularly in hospital-based settings where coordination, continuity, and care transitions are essential.

Furthermore, existing literature has generally focused either on advanced nursing roles broadly or on integrated care as a general concept, with limited attention to the specific contributions of GNSs in supporting integrated care for older adults. Consequently, there remains a lack of synthesized evidence clearly describing the roles, functions, and practical activities of GNSs within this context. This gap is particularly important given the increasing complexity of older adults’ care needs and the important role of hospitals in initiating and coordinating care processes.

Given the breadth of the topic, variability in role definitions, and the diversity of study designs and healthcare contexts, a scoping review is considered an appropriate methodological approach. Scoping reviews are particularly suitable for mapping key concepts, clarifying definitions, and identifying gaps in the existing literature, especially where evidence is heterogeneous and not yet comprehensively synthesized [25,26].

Therefore, this scoping review aims to systematically map the roles of GNSs in supporting integrated care for older adults in hospital-based settings, examine how these roles are conceptualized and enacted in practice, and identify gaps in the current evidence.

## 2. Review Question

The overarching research question of this scoping review is ‘What roles do GNSs play in supporting integrated care for older adults within hospital-based settings?’ The sub-questions are as follows:

(1) How do the roles of hospital-based GNSs in supporting integrated care vary across different healthcare systems, policies, and sociopolitical or sociocultural contexts?
(2) What functions, responsibilities, and activities underpin these roles?
(3) What gaps exist in the current evidence regarding the roles of GNSs in supporting integrated care for older adults?

## 3. Materials and Methods

This scoping review will be conducted in accordance with the Joanna Briggs Institute (JBI) methodology for scoping reviews [25]. The eligibility criteria for study selection will be developed according to the Participants, Concept, and Context (PCC) framework and types of sources recommended by JBI methodology. This protocol has been registered with the Open Science Framework (OSF) (https://osf.io/vpshm, accessed on 19 May 2026). The final scoping review will be reported in accordance with the Preferred Reporting Items for Systematic Reviews and Meta-Analyses extension for Scoping Reviews (PRISMA-ScR) [27].

### 3.1. Inclusion Criteria

#### 3.1.1. Participants

This review will consider studies involving GNSs, defined as nurses with recognized specialist or advanced nursing practice competence in gerontological nursing or older-adult care. This may include CNSs, APNs, NPs, and equivalent gerontological specialist nursing roles described in the literature. Studies involving nurses providing care to older adults without a clearly described gerontological specialist or advanced practice role will be excluded.

#### 3.1.2. Concept

The concept of this review is the roles of GNSs in supporting integrated care for older adults. This includes how such roles are conceptualized, defined, operationalized, and implemented across different healthcare systems, policies, and sociopolitical or sociocultural contexts, as well as the functions, responsibilities, and activities associated with these roles in promoting coordinated, continuous, and person-centred care across providers and services. Relevant activities may include, but are not limited to, comprehensive assessment, care coordination, interdisciplinary collaboration, discharge planning, care transition support, patient and family education, referral and follow-up.

#### 3.1.3. Context

The context of this review includes hospital-based settings and hospital-linked transitional care services for older adults. This encompasses acute care hospitals and related services where integrated care processes are initiated, coordinated, or supported, including geriatric wards, geriatric consultation services, discharge planning or support services, and post-discharge follow-up services. Studies conducted solely in community-based, primary care, residential aged care, or long-term care settings will be excluded unless they explicitly involve hospital-initiated, hospital-coordinated, or hospital-linked care processes or transitions.

#### 3.1.4. Types of Sources

This scoping review will consider a range of evidence sources, including quantitative, qualitative, and mixed-methods studies. Primary research studies will constitute the main sources of evidence. Systematic reviews and scoping reviews will be considered primarily to identify additional relevant studies through reference list screening but will not be included in the formal data extraction unless they provide unique evidence not available in the primary studies. In addition, relevant policy documents, clinical guidelines, and expert consensus statements will be considered where they explicitly describe the roles, functions, responsibilities, and activities of GNSs.

#### 3.1.5. Search Strategy

The search strategy will aim to locate both published and relevant non-research documents that meet the inclusion criteria. A three-step search approach will be employed in accordance with JBI methodology for scoping reviews [25].

First, an initial limited search of PubMed and CINAHL was conducted on 25 May 2026 to identify relevant articles. Key search concepts included: (1) GNSs and related specialist nursing roles (e.g., APNs, CNSs, NPs); (2) nursing roles, functions, responsibilities, and activities; and (3) integrated care and related care processes (e.g., care coordination, transitional care, collaborative care). The text words contained in the titles and abstracts of relevant articles, as well as the index terms used to describe the articles (e.g., MeSH terms in PubMed and CINAHL subject headings), were analyzed to inform the development of a comprehensive search strategy for each database (Table 1). Both controlled vocabulary and free-text terms were used to maximize the sensitivity and comprehensiveness of the search strategy.

Second, the identified keywords and index terms will be used to conduct a comprehensive search across multiple databases. English-language databases will include PubMed, CINAHL (via EBSCOhost), and Web of Science. Japanese-language studies will be searched in Ichushi-Web, and Chinese-language studies will be searched in China National Knowledge Infrastructure (CNKI). The search strategies for Ichushi-Web and CNKI were developed using database-specific controlled vocabulary (where available) and free-text terms appropriate to the indexing systems and search functions of each database, rather than through direct translation of the English-language search strategy. The Japanese and Chinese search terms were iteratively refined through pilot searches to optimize retrieval performance before the final search strategies were established. No date restrictions will be applied.

In addition, a targeted grey literature search will be undertaken to identify relevant policy documents, clinical guidelines, and expert consensus statements. Grey literature sources will be identified through the websites of international organizations (e.g., the World Health Organization and the International Council of Nurses), governmental health agencies, and relevant professional nursing organizations. Search terms will be adapted to the terminology and search functions of each website and will combine terms related to gerontological nurse specialists, integrated care, older adults, and nursing roles. Grey literature sources will be screened against the same eligibility criteria as published studies and included only if they explicitly describe the roles, functions, responsibilities, or activities of hospital-based GNSs in supporting integrated care.

Third, the reference lists of all included studies will be reviewed to identify additional potentially relevant studies that may have been missed during the database search. Citation searching will also be conducted where appropriate. A university librarian with expertise in systematic review searching was consulted during the development of the search strategies, and they reviewed and refined the final search strategies, including the database-specific search terms, controlled vocabulary, and indexing approaches.

### 3.2. Source of Evidence Selection

All retrieved records will be imported into EndNote 21 (Clarivate Analytics, Philadelphia, PA, USA) for management, and duplicates will be removed.

Prior to the formal screening process, two reviewers will independently conduct a pilot screening of a random sample of 10% of the titles and abstracts. The pilot screening results will be discussed in a consensus meeting to ensure a shared understanding of the eligibility criteria and to refine the criteria if necessary. Formal screening will begin once at least 75% agreement between reviewers has been achieved [28].

Following the pilot screening, two reviewers will independently evaluate the remaining titles and abstracts against the inclusion criteria. For studies that meet the inclusion criteria, or for which eligibility is unclear, full text will be retrieved. The full-text articles will then be assessed independently by the two reviewers. Reasons for exclusion at the full-text stage will be recorded and reported in the final review. Any disagreements between reviewers at each stage of the selection process will be resolved through discussion. If consensus cannot be reached, a third reviewer will be consulted. The results of the search and the study selection process will be reported in full in the final scoping review and presented using a PRISMA-ScR flow diagram in accordance with reporting guidelines.

### 3.3. Data Extraction

Data will be extracted from included studies by the two independent reviewers using a data extraction form developed specifically for this review. Prior to full data extraction, the extraction form will be pilot tested on a sample of included studies to ensure consistency and comprehensiveness and may be refined as necessary during the review process. Microsoft Excel will be used to organize and manage the extracted data.

The preliminary data extraction instrument is presented in Table 2. Data extraction will include evidence source characteristics, contextual characteristics (e.g., healthcare system, policy, and sociopolitical or sociocultural context, if reported), and information related to the roles of GNSs in supporting integrated care for older adults. These contextual data will facilitate comparative synthesis and interpretation of GNS roles across different healthcare systems and sociopolitical or sociocultural contexts. Any disagreements between reviewers during the data extraction process will be resolved through discussion or consultation with a third reviewer.

### 3.4. Data Analysis and Presentation

The extracted data will be analyzed using a descriptive analytical approach in accordance with the JBI methodology for scoping reviews. The analysis will focus on systematically organizing, summarizing, and mapping the available evidence regarding the roles of GNSs in supporting integrated care for older adults in hospital-based settings.

Data related to role titles, role definitions and conceptualizations, functions, responsibilities, and activities will be organized and categorized according to similarities and differences across the included studies. These categories will be used to synthesize how GNS roles are conceptualized and enacted in the literature. Where reported, contextual characteristics (e.g., healthcare systems, policy, and sociopolitical or sociocultural context) will also be synthesized to facilitate comparative interpretation of GNS roles across different settings. In addition, gaps in the current evidence regarding GNS roles in supporting integrated care for older adults will be identified and summarized descriptively. The categorization and interpretation processes will be discussed among the reviewers to enhance analytical rigor and consistency. Any disagreements will be resolved through discussion and consultation with a third reviewer where necessary.

The findings will be presented using tabular, narrative, and diagrammatic formats. Summary tables will be used to present study characteristics, role titles, role definitions, and identified functions, responsibilities, and activities. Where supported by the evidence, the synthesized findings will also be presented according to overarching role domains, integrated care functions, and specific professional activities to facilitate comparison and interpretation across studies. The geographical distribution and contextual characteristics of the included studies will also be summarized descriptively to facilitate interpretation of the findings across different settings. Conceptual mapping will be used to illustrate the relationships between GNS roles and integrated care components, such as care coordination, transitional care, interdisciplinary collaboration, and continuity of care. A narrative summary will accompany the tables and figures to explain how the findings address the review objectives and research questions.

## 4. Expected Contributions

This scoping review is expected to address an important gap in the literature regarding the roles of GNSs in supporting integrated care for older adults within hospital-based settings. Given the diversity of professional titles, role definitions, healthcare systems, and sociopolitical or sociocultural contexts across countries, the contributions of GNSs remain inconsistently described and insufficiently synthesized.

By systematically mapping the role conceptualizations, functions, responsibilities, activities, and contextual characteristics reported in the literature, this review is expected to identify key role domains and provide a comprehensive understanding of how GNSs support integrated care for older adults across different healthcare systems. The findings are expected to inform future research, support role clarification, and contribute to workforce development, nursing education, and the development of integrated care models and policies in gerontological nursing.

## 5. Conclusions

As a scoping review, this study is expected to provide a comprehensive overview of the existing evidence rather than evaluate the effectiveness of GNS roles. Potential limitations include the restriction to studies published in English, Chinese, and Japanese, which may result in the omission of relevant evidence published in other languages, as well as the anticipated heterogeneity of GNS role definitions and healthcare contexts across countries, which may limit direct comparisons between studies.

## Figures and Tables

**Table 1:** Search strategy for PubMed, conducted on 25 May 2026.

Search	Query	Records Retrieved
#1	“Advanced Practice Nursing”[MeSH] OR “Nurse Practitioners”[MeSH] OR “Nurse Clinicians”[MeSH] OR “Geriatric Nursing”[MeSH] OR “Nurse Specialists”[MeSH] OR advanced practice nurse*[tiab] OR nurse practitioner*[tiab] OR clinical nurse specialist*[tiab] OR advanced practice registered nurse*[tiab] OR certified nurse specialist*[tiab] OR gerontolog* nurse*[tiab]	65,189
#2	“Nurse’s Role”[MeSH] OR role*[tiab] OR function*[tiab] OR responsibilit*[tiab] OR activit*[tiab] OR practice*[tiab]	12,078,663
#3	“Delivery of Health Care, Integrated”[MeSH] OR integrated care[tiab] OR care coordination[tiab] OR coordinated care[tiab] OR transitional care[tiab] OR collaborative care[tiab] OR continuity of care[tiab]	50,591
#4	“Aged”[MeSH] OR older adult*[tiab] OR older people[tiab] OR older person*[tiab] OR elderly[tiab] OR geriatric*[tiab] OR aging[tiab] OR ageing[tiab]	4,283,648
#5	#1 AND #2 AND #3 AND #4	239

**Table 2:** Data extraction instrument.

Evidence Source Details and Characteristics
Bibliographic information (author(s), year of publication, title, journal)
Country/region
Contextual characteristics (e.g., healthcare system, policy, sociopolitical or sociocultural context, if reported)
Study design
Study setting
Participants
Details/Results extracted from source of evidence
Type of GNS roles (role titles, role definitions or conceptualizations)
Integrated care model or context
Functions, responsibilities, and activities
Role-related findings (e.g., clinical assessment, care coordination, discharge planning, transitional care, patient and family education, interprofessional collaboration, leadership, consultation, and other role-related activities, where applicable)

## Data Availability

The data presented in this study are all available in this manuscript.
